# Types of T-cell lymphoma-a cytogenetic perspective

**DOI:** 10.1016/j.amsu.2022.104844

**Published:** 2022-11-09

**Authors:** Muhammad Ammar Samad, Eman Mahboob, Aimen Shafiq, Mohammad Hassam Ur Rehman, Ayesha Sheikh, Zoaib Habib Tharwani

**Affiliations:** Faculty of Medicine, Dow Medical College, Dow University of Health Sciences, Karachi, Pakistan

**Keywords:** T-cell lymphomas, Cytogenetics, Mutations, Non-hodgkin lymphomas

## Abstract

T cell lymphoma, a type of non-Hodgkin lymphomas is a rare form of malignancy with poor outcomes.

TCLS are a heterogeneous group of lymphoid malignancies that occur in nodal and extranodal sites.

There are two main types of TCLs namely T-lymphoblastic lymphoma/leukemia and Peripheral T-cell lymphomas classified based on clinical manifestations and cytogenetic mutations.

The use of advance technology like karyotyping, fluorescent in situ hybridization (FISH), comparative genomic hybridization (CGH) has allowed us to study TCLs in detail and to observe a different biochemical change that occurs in different TCLs allowing us to classify and treat them differently.

This review focuses on the different mutations occurring in different TCLs and how they help us distinguish one type from another.

## 1 Introduction

T-cell lymphoma is a type of non-Hodgkin lymphoma (NHL), which is a cancer that originates in the lymph tissue. Lymphoma occurs when certain gene changes, called mutations, form within white blood cells. Based on characteristic clinical manifestations, cytogenetical changes and major cell lineage involved T cell lymphomas can be broadly classified into T-lymphoblastic lymphoma/leukemia and Peripheral T-cell lymphomas. In this article we focus on cytogenetical differences in TCLs. Due to its broad classification, each with different characteristics establishing a better treatment for TCLs have been challenging but with the help of advance techniques like karyotyping, fluorescent in situ hybridization (FISH), comparative genomic hybridization (CGH) we are now able to study different cytogenetical changes occurring in different types of TCLs and distinguish one type from other. The different mutations are highlighted and compared in the article which provides a better insight to as to what we are dealing with and assist us to come up with improved medical interventions.

However, it's unclear why this happens or whether it's hereditary. Since there are many types of T-cell lymphoma, certain risk factors may be more relevant for one type than another.

## 2 T-cell lymphoblastic lymphoma (T-LBLL)

T-LBLL, a type of non-Hodgkin lymphoma is a rare cancer in which there is aggressive proliferation of immature lymphoblasts in the hematopoietic stem cells [1].

Almost ninety percent of the lymphoblastic lymphomas (LBL) are T-LBL and predominantly occur in young children, most of which are males rather than females [2].

The detailed mechanisms of the molecular patterns of T-LBL are still being worked on, but some known genetic markers are mentioned below [3].

The modified T-cell transforming genes most commonly lead to genetic abnormalities in the TCR- α and TCR-δ genes site, in the region 14q11-13 [[4], [5], [6]]. Also the new arrangements between TCR-β and TCR-γ genes sites is also seen in patients suffering from T-LBL [4].

Chromosomal translocations, deletions, pseudodiploid karyotypes in the 9q34 region are seen as regular patterns [7].

Mutations like NOTCH1 have been found in children with T-LBL. It has been proved by many recent studies (3) that NOTCH1 mutant patients have a greater survival rate as compared to patients who lack this mutation [8]. Also, the cancer reoccurrence rate was shown to be low in patients having NOTCH1 mutation [9]. Although NOTCH1 mutations itself have propitious results in patients with T-LBL but studies have shown a non-favorable outcome in NOTCH1 mutant patients having increased expression of *miR-223* levels [10].

Loss of heterozygosity i.e., the deletions in the long arm of chromosome 6 have been associated with T-LBL. Chromosome 6 have some important tumor suppressor genes and hence deletions can lead to malignancies. LOH6q positive patients have a poor prognosis and greater reoccurrence chances [11] as it effects several genes like the caspase 8 associated protein 2 gene (*CASP8AP2*/*FLASH*) which plays a role in FAS mediated apoptosis, glutamate ionotropic receptor kainate type subunit 2 gene (*GRIK2) and* tyrosine kinase receptor gene ephrin type-A receptor 7 (*EPHA7*). Although the clear mechanism of these gene mutations is still unknown, they play an important role in the pathogenesis of T-LBL [[12], [13], [14]].

Another gene mutation *PTEN* have been found to have a poor prognosis in children with T-LBL. It has also been proved that *PTEN* mutant patients who were LOH6q positive had the worst outcomes [15]. On the other hand, *NOTCH1* mutant patients having *PTEN* mutated gene had a much better prognosis, as the favorable effects of *NOTCH1* antagonizes the unfavorable effects of *PTEN* mutations [23]. Other pediatric T-LBL mutation genes include *PIK3R1*, *PIK3CA.* [15].

Thymic T-cells require some essential cytokines like Interlukin-7 (IL7) for maturations and development. [15, 16, 17, 18,19] IL7 signaling activates different downstream pathways like Janus kinase 1 (JAK1), JAK3 and phosphoinositide 3−kinase (PI3K). [20, 21, 22] Mutations in IL7, JAK1, JAK3 have been reported in many adult patients with T-LBL [3].

Mutations in *PHF6* gene responsible for epigenetic modifications have been seen T-LBL patients but have a positive prognosis [3].

More studies are needed for a clear picture of genetic mechanisms in patients with T-LBL.

## 3 Peripheral T-cell lymphomas (PTCL)

Peripheral T-cell lymphoma (PTCL) is a rare and heterogeneous group of clinically aggressive diseases associated with poor prognosis. Except for ALK + anaplastic large-cell lymphoma (ALCL), most peripheral T-cell lymphomas are highly malignant and have an aggressive disease course and poor clinical outcomes, with a poor remission rate and frequent relapse after first-line treatment [24].

PTCLs are further divided into subtypes:

i) Cutaneous T-cell lymphoma (CTCL):

The two common types of CTCL are mycosis fungoides (MF), consisting of more than half the cases of CTCL (approx. 55%) and Sezary syndrome (SS) the rarer type comprising of around 5% cases [25,26].

MF having a longer clinical course presents in sun protected areas with patches and can eventually develop into a tumour compromising other organs leading to the immunosuppression of the person [27].

SS on the other hand presents with inflammation of the skins surface with swelling of lymph nodes and spread of malignant CD4^+^ T cells to the peripheral blood making it a more aggressive tumour than MF. Recurrence is more common in SS and generally has a less survival rate than MF [[28], [29], [30]].

Karyotypic, cytogenetic, and array comparative genomic hybridization studies have shown different chromosomal abnormities in different CTCLs.

In tumour MF the most common mutation is the loss of 9p21 and the addition of 1p, 1q, 7q, and 8/8q and this mutation is linked with poor outcome in MF.

There is an increase in Microsatellite instability in MF and this is seen in 24% of CTCL cases. In SS there is structural and numerical mutations most commonly involving chromosomes 6 and 10 although chromosomes 3, 7, 9, 17, 1, 12, 8, 11, and 13 can also be involved [[31], [32], [33]].

The mutations noted in SS in different patients are non-recurring unbalanced translocation hence There are no specific defining mutations in SS [34]. Using FCA SS can be distinguished from benign dermatoses as SS have a greater proportion of CD41/CD7 cells. MF however doesn't show any increase in either cell type [35].

FCA could detect aberrant CD2, CD3, or CD5 in 66% of SS and 30% of MF patients.

Other gain of function mutations seen in CTCL include Nav3 (12q21), JunB (chr19), c-MYC/MAX, p53, PTEN/Fas, p15, p16, NFKB, bcl-2, and Stat2.The mutations in CTCL are still being studies and as of now there are more than 500 chromosomal abnormalities, including TCR signalling and chromatin modification, noted in CTCL.

Loss of expression of total t cell marker in more than one-fourth cells and loss of antigens on more than half of t cells is alarming for T cell LPD as is the case with other mature T cells lymphomas.

These findings are found to be 78% sensitive and 89% specific in comparison to non-MF or indeterminate histologies [36].

ii) Adult T-cell Lymphoma (ATL):

Adult T-cell Leukemia/Lymphoma (ATL) is a unique neoplasm of peripheral T-lymphocytes driven by human T-cell lymphotropic virus type 1 (HTLV-1) [37,38]. Nearly 2–3% of women and 6–7% of men HTLV-1 carriers suffer from ATL after a lag phase of 30–50 years following infection. HTLV-1 spread occurs predominantly through lactation. These findings imply that, despite the crucial roles that HTLV-1-derived proteins like HBZ and Tax perform in the pathogenesis of ATL, further epigenetic and/or genetic changes are necessary for HTLV-1-infected cells to develop into ATL [38].

In ATLL, the karyotype is complicated and devoid of any recognizable abnormalities, especially in the acute forms. Monosomy X, 3p aberrations, deletion of the Y chromosome, chromosomal anomalies (+3, +7, +21, X, Y), and translocations affecting the 14q32 and 14q11 regions of the TCR (delta) genes and TCR (alpha), respectively, are included in the irregularities associated with ATLL [39], [43]. Unlike FOXP3 ATLLs, FOXP3+ ATLLs exhibit fewer complicated cytogenetic aberrations. It is suggested that a mixed karyotype, which is regular at the initial diagnosis, is more frequently detected in ATLL recurrence, highlighting the idea of multicomponent lymphomagenesis in ATLL/[40].

The T-cell receptor/NF–B signaling pathway is frequently altered in ATLL [44], leading to genetic alterations in the PRKCB, PLCG1, VAV1, and CARD11 genes as well as the ICOS-CD28 and CTLA4-CD28 fusions. Additionally, immune regulatory molecules such as CD58, HLA-A/B, and FAS are frequently impacted. Moreover, The PD-L1 3′-untranslated domain is one of the most frequently truncated by structural changes among them, which causes overexpression. Additional genetic markers comprise transcription factors (IKZF2, IRF4, and GATA3) and chemokine receptors (GPR183, CCR4, and CCR7), which have clinical significance in healthy T cells. About 50% of ATLL cases have been shown to have deletions or mutations of the tumor suppressor genes CDK2B (p15), CDKN2A (p16), and TP53 (p53), and this result may guide future treatments [39], [42].

Almost all ATLL episodes include monoclonal HTLV-1 provirus DNA insertion, which can be detected by polymerase chain reaction or Southern blotting [41]. A diagnosis can be made with just a positive HTLV-1 serological test and the relevant morphologic and clinical features of ATLL. When there is a strong suspicion of ATLL in a seronegative individual, HTLV-1 molecular testing is needed [42].

iii) Angioimmunoblastic T-cell lymphoma (AITL):

Angioimmunoblastic T-cell lymphoma (AITL) is a subtype of mature peripheral T-cell lymphoma (PTCL) and is recognized since the 1994 Revised European American Lymphoma (REAL) Classification [45,46]. AITL is a systemic disease that classically presents with B symptoms (fevers, severe weight loss, and/or profuse night sweats), polyadenopathy, and numerous immunologic anomalies which include elevated sedimentation rate leading to positive autoimmune tests such as rheumatoid factor, anti-smooth muscle, and with circulating immune complexes or cold agglutinins [47]. The biopsy of the lymph node in AITL is usually diffusely polymorphous infiltrate Similar to the Reed-Sternberg cells of Hodgkin lymphoma, the malignant TFH cells in AITL represent a minority of the cellular components of a malignant lymph node. The architecture of the lymph node is commonly effaced and devoid of follicles [47], the rapid proliferation of follicular dendritic cells (FDCs), and the presence of positive large B-cell blasts that correspond to Ebstein Barr virus (EBV) [48]. Markers such as CD200, NFATC1, PDCD1, CD40L, and LIF are overexpressed in THF cells thus supporting the AITL derivation from TFH cells [49].

**Fig. 1:**
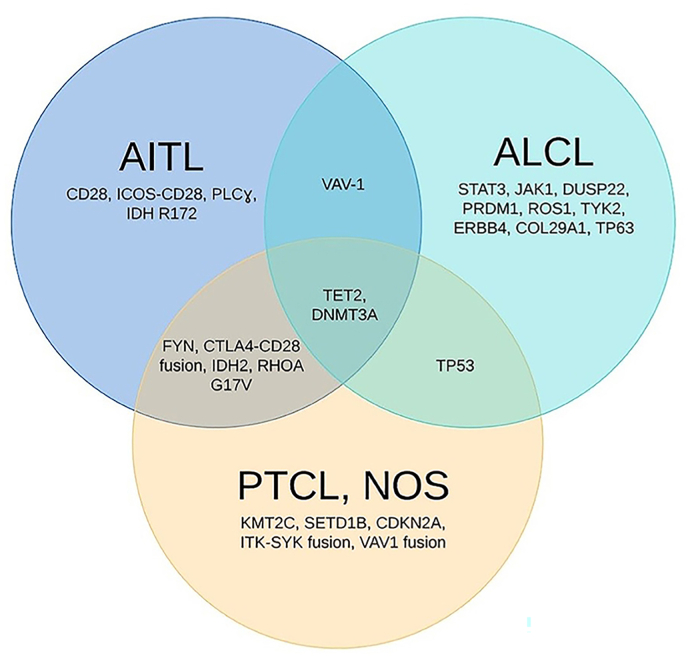
Genetic abnormalities in Angioimmunoblastic T-cell lymphoma (AITL), Anaplastic Large cell Lymphoma (ALCL) and Peripheral T-cell lymphoma, not otherwise specified (PTCL, NOS). From “Genomics of Peripheral T-Cell Lymphoma and Its Implications for Personalized Medicine” by Zhang Y 2020, Frontiers in oncology, 10, 898. https://doi.org/10.3389/fonc.2020.00898.

Genes like CD10, BCL6, PD1, CXCL13, CCR5, SAP, and ICOS are markers considered sufficient to suggest the derivation of a given T-cell neoplasm from TFH for AITL and as well as for PTCL NOS as shown in Fig. 1 [50]. Recent research and studies have shown that AITL is caused by mutation of multiple genes that correspond to b cell diseases, e.g., loss-of-function mutations in TP53, ETV, CCND3, and EP300 as gain-of-function mutations in JAK2 and STAT3. the most frequently mutated genes in AITL include TET2, DNMT3A, and IDH2 [51].

## 4 Extra nodal natural killer/T-cell lymphoma, nasal type (ENKTL/NKTCL)

Natural killer T-cell lymphoma (NKTCL), previously known as Extra nodal natural killer T-cell lymphoma (ENKTL), nasal type, is an uncommon disease that primarily affects Asians. It is characterised by extra nodal involvement and is closely linked to the Epstein-Barr virus (EBV). [52, 53]Difficulty to discriminate between cell lineages using typical immunohistochemical markers, ENKTL covers both NK-cell (85%) and T-cell lymphomas (15%). [54,55] Most NK-cell malignancies, especially the more advanced ones, exhibit cytogenetic abnormalities. In the detected karyotypes more than half were pseudodiploid, around one-third hyper diploid and 13% showed hypodiploidy [56]. Chromosome 6q (contain the potential tumour suppressor genes PRMD1, HACE1, FOXO3, and PTPRK), 11q, 13q, and 17p deletion were most frequently observed cytogenetic aberrations by fluorescent in-situ hybridization (FISH), which is primarily utilised to detect deletion of chromosome 6q21–25 along with i(6)p(10). [57,58] Molecular investigation of clinical samples, revealed that several tumour suppressor genes, including p16NK4A, p15NK4B, p14ARF, TP53 and Rb have been rendered inactive., coupled with changes in FAS, B-catenin, and KIT [59]. Significant quantities of methylation are present at the epigenetic level in several NK-cell related and tumour suppressor genes that includes: p15 (48%), RARbeta (56%), hMLH1 (61%), p16 (71%), and p73 (92%) [60]. Gene expression profiling (GEP) had identified additional carcinogenic mechanisms that contribute to NK/T-cell lymphomas, such as upregulation of aurora kinase A and NK-B and stimulation of the JAK/STAT pathway [61, 62].

## 5 Enteropathy-associated T-cell lymphoma (EATL)

Enteropathy-associated T-cell lymphoma (EATL) is an uncommon but extremely severe gastrointestinal tumour of intraepithelial T lymphocytes, included in the category of mature T cell non-Hodgkin lymphomas [63, 64]. Subcategories of EATL include: Type I which has a substantial but not exclusive link to celiac disease (CD). Morphologically similar small-to medium-sized masses are the hallmark of Type 2 EATL, commonly known as monomorphic epitheliotropic intestinal T cell lymphoma (MEITL), which has no relation with CD [65]. According to extensive research, all kinds of EATL exhibits complicated amplification of 9q33-q34 and losses of 16q12.1 [66]. The 1q and 5q chromosomes generally exhibits amplification in type 1 EATL [67], while the majority of monomorphic EATL patients display an upregulated 8q24, that is the c-MYC oncogene locus. The c-MYC protein contributes to the apoptotic pathway and the growth of lymphocytes in neoplastic lymphomas, suggesting that c-MYC is a key transcription factor [68]. In a study using microsatellite markers, Baumgartner et al. observed that EATL-2 was linked to reduced amounts of microsatellite instability, propensity for less allelic instabilities at several loci and 3q27 allelic imbalances [69]. A decrease of heterozygosity at 9p21 (targeting CDKN2A/B) was seen in more than half of the cases studied [70]. The aforementioned findings were supported by a study from Japan, that showed several chromosomal abnormalities that included: gains in 9q,7q, 1q and 5q and losses in 8p, 9p, and 13q [71] The most often dysregulated pathway was the JAK-STAT one, which had various mutations in SOCS1, STAT3, STAT5B, JAK1 and JAK3 [66].

## 6 Anaplastic large cell lymphoma (ALCL)

The term “anaplastic large cell lymphoma” (ALCL) was initially used to characterize a large-cell neoplasm having an anaplastic architecture that was marked by the CD30-specific Ki-1 antibody. As a result of the discovery of the nucleophosmin (NPM)-anaplastic lymphoma kinase (ALK) fusion receptor tyrosine kinase in a subgroup of patients, this illness is now classified by the World Health Organization as either ALCL that is ALK-positive or -negative.

i) ALK-positive ALCL:

ALK + ALCL exhibits clonal alteration of the TCR genes in about 90% of cases [78]. The ALK gene on chromosome 2 and the nucleophosmin (NPM) gene on chromosome 5 are translocated, or t (2;5) (p23; q35), to cause the majority of genetic changes [72, 73, 74]. ALK and other companion genes on chromosomes 1, 2, 3, 17, 19, 22, and X also experience various genetic recombination. ALK is upregulated as a result of each of these genetic alterations. Epstein-Barr virus testing is routinely negative with ALK + ALCL [75]. Recurrent increments of 17q24 and 17p and deficits of 11q14 and 4q13-q21 were found in ALK + ALCL reports [76]. The top four genes out of the total 117 that were overexpressed in ALK + ALCL were PTPN12 (tyrosine phosphatase), BCL6, C/EBP, and serpinA1. Recently, it was shown, by ALK or STAT3 GEP signatures acquired from ALK + ALCL cell cultures that predominant central ALK + ALCL displays a unique profile, mostly controlled by STAT3 signaling [77].

ii) ALK-negative ALCL:

Most ALK-ALCL exhibit observable TCR gene alteration, similar to ALK + ALCL (78). TNFRSF8, TMOD1, and GATA3 are among the genes whose expression is predominant in ALK-negative ALCL and is mediated by PI3K signaling. Frequent gene rearrangements are observed in a small percentage of cases, with the most prevalent translocation (30%) involving readjustment of the DUSP22 locus (6p25.3), which encrypts a dual specific phosphatase with tumor suppressor action. The most frequent translocation is t (6;7) (p25.3; q32.3), which transcribes the DUSP22 locus on chromosome 7 [79]. Only in the instances with ALK-ALCL are alterations in the musculin gene (MSC) observed, and in 93% of these instances, DUSP22 translocations are also present [80]. Reallocation of the TP53 homolog, TP63 (3q23), which is seen in about 8% of patients and most usually takes the shape of (3) (q26q28), is another prevalent change that is thought to be correlated with an especially bad prognosis. Numerous chromosomal additions and deletions (5q, 1q41-qter, 8q, 6p, 17q, and 12q) are other genetic changes (4q, 6q21, and 13q21-q22) [81].

By using next-generation genotyping, frequent gene alterations are found in about 18% of cases of ALK-ALCL. The JAK-STAT signaling system is constitutively activated as a result of the most often mutant genes, STAT3 and JAK1. TP53, PRDM1, FAS, TET2, and STIM2 were among the genes that were less often mutated. It's noteworthy that phosphorylated STAT3 was discovered in cases lacking gene alterations, pointing to potential alternative JAK-STAT activation pathways. According to cytogenetic research, a subgroup of ALK-ALCL has elevated ERBB4 gene transcription.

## 7 Peripheral T-cell lymphoma, not otherwise specified (PTCL, NOS)

PTCL NOS, a diagnosis of exclusion is also known as wastebasket It accounts for 30%–35% of all PTCL and is considered the most common subtype globally [82]. like most PTCL, it is mostly nodal lymphoma with extra-nodal involvement that includes skin, lungs, gastrointestinal tract, and bone marrow. It is one of the most aggressive and chemo-resistant neoplasms with a 5-year overall survival rate [83]. The cytology in PTCL-NOS is often pleomorphic, and like AITL it consists of a mixed population of medium to large cells with a rapid proliferation rate with Clear cells. [84]. This neoplasm cytogenic involvement also resembles AITL with mutations that are homogeneous (TET2, IDH2, DNMT3A, RHOA, and CD28) as shown in Fig. 1. Because of this similarity in both AITL and PTCL NOS extensive immunophenotyping is required to distinguish between them [85]. One study reported that 37% of PTCL-NOS cases were reindexed as AITL and about 22% of AITL cases were reindexed as PTCL-NOS based on GEP and genetic studies [86, 87]. Recent studies to better classify PTCL NOS have divided it into two subgroups based on prognosis, out of which one is characterized by over-expression of GATA-binding protein 3 (GATA3) while the second one is T-box 21 (TBX21) and eomesodermin (EOMES). The target genes of which are CCR4, IL18RA, CXCR7, IK and CXCR3, IL2RB, CCL3, and IFNc respectively. [86], [88, 89]).

## 8 Future implications and limitations

Although TCLs account for less than 15% of all NHLs, they are usually aggressive and can occur in the lymph nodes, skin (cutaneous T-cell lymphomas), or other parts of the body (peripheral T-cell lymphomas). The rate of survival in patients, specifically adults who are diagnosed with T-cell lymphomas is 63.1% whereas patients with NHL excluding TCLs have a higher survival rate of 66.9%. Moreover, TCLs can lead to long term side effects like leading to other cancer types, and other conditions involving other major organs of the body.

Taking into account the above statistics various treatment options are being explored for each type of TCL such as, multiple targeted therapies for aggressive kinds of TCLs like PTCL have been established against multiple receptors expressed on tumor surfaces like Brentuximab vedotin, for CD30 receptor, ALK inhibitors for ALK expressing tumors and other combinational chemotherapies against different receptors like CCR4, CD25 CD38, PI3Kinase and JAK/STAT are being explored [90]. Other curatives like interferons, photopheresis, retinoids have been helpful in managing CTCL, in addition to that immune cell therapies like CAR-T, IL-12, TLR-agonists have showed encouraging effects in patients with CTCL during various clinical trials [91]. Similarly, chemotherapies like intensive remission induction, central nervous system (CNS) prophylaxis and consolidation chemotherapy have been developed for T- Cell Lymphoblastic Lymphoma (T-LBL) and although the patients have shown a higher response rate to these managements, the overall rate of survival remains low [92].

We need more trials which focus on the different biomarkers, transcriptional factors, cytopathological and clinical advancement of TCLs so that we can come up with an out of the box approach for the treatment and management and hence basic knowledge like that of cytogenetics can help us conduct different clinical researches and develop less toxic cures and hence increase the rate of survival.

Furthermore, the limitations in the current data on the cytogenetic classification of T-cell lymphomas should be taken into consideration in order to derive a non-biased conclusion about them. It is difficult to determine the clinical importance of secondary chromosomal abnormalities because the same aberration may have been proven to be significant for prognosis in one subtype of T-cell lymphoma but not in another. Since most cytogenetic research on T-cell lymphomas only covered a small number of patients, the frequency of specific aberrations in each distinct subtype has varied greatly between investigations. The use of various classification methods, treatment protocols, and even geographic diversity can result in some discrepancies between studies. Additionally, numerous investigations have been carried out after therapy, introducing anomalies that might be brought on by the medication instead of the primary disease process.

## 9 Conclusion

Different types of TCL present with different symptoms, in different age groups some affecting one gender more than the other. Whilst all TCLs are type of non-Hodgkin lymphomas there are different mutations seen in different TCLs allowing us to distinguish them apart.

Cytogenetics advances have helped us greatly in this regard and recent developments in molecular genetic findings have shown the potential for further improvements in our understanding of TCL and its treatment.

## **Ethical** approval

N/A.

## Sources **of** funding

None.

## **Author** contribution

All authors equally contributed.

## Registration **of research**

1 Name of the registry: N/A
2 Unique Identifying number or registration ID: N/A
3 Hyperlink to your specific registration (must be publicly accessible and will be checked): N/A

## Guarantor

N/A.

## Consent

N/A.

## Provenance and peer review

Not commissioned, externally peer reviewed.

## Declaration of competing interest

None declared.

## References

[1] https://www.cap.org/member-resources/case-of-the-month/t-lymphoblastic-leukemia-lymphoma-tlbll-2019.

[2] Lepretre S., Graux C., Touzart A., Macintyre E., Boissel N. (2017). Adult T-type lymphoblastic lymphoma: treatment advances and prognostic indicators. Exp. Hematol..

[3] Li Z., Song Y., Zhang Y., Li C., Wang Y., Xue W., Lu L., Jin M., Zhou Z., Wang X., Li L., Zhang L., Li X., Fu X., Sun Z., Wu J., Zhang X., Yu H., Nan F., Chang Y., Zhang M. (2020). Genomic and outcome analysis of adult T-cell lymphoblastic lymphoma. Haematologica.

[4] Bassan R., Maino E., Cortelazzo S. (2016). Lymphoblastic lymphoma: an updated review on biology, diagnosis, and treatment. Eur. J. Haematol..

[5] Cortelazzo S., Ponzoni M., Ferreri A.J., Hoelzer D. (2011). Lymphoblastic lymphoma. Crit. Rev. Oncol.-Hematol..

[6] Baleydier F., Decouvelaere A.V., Bergeron J., Gaulard P., Canioni D., Bertrand Y., Lepretre S., Petit B., Dombret H., Beldjord K., Molina T., Asnafi V., Macintyre E. (2008). T cell receptor genotyping and HOXA/TLX1 expression define three T lymphoblastic lymphoma subsets which might affect clinical outcome. Clin. Cancer Res. : off. j. Am. Assoc. Cancer Res..

[7] Sekimizu M., Sunami S., Nakazawa A., Hayashi Y., Okimoto Y., Saito A.M., Horibe K., Tsurusawa M., Mori T. (2011). Chromosome abnormalities in advanced stage T-cell lymphoblastic lymphoma of children and adolescents: a report from Japanese Paediatric Leukaemia/Lymphoma Study Group (JPLSG) and review of the literature. Br. J. Haematol..

[8] Bonn B.R., Rohde M., Zimmermann M., Krieger D., Oschlies I., Niggli F., Burkhardt B. (2013). Incidence and prognostic relevance of genetic variations in T-cell lymphoblastic lymphoma in childhood and adolescence Blood. J. Am. Soc. Hematol..

[9] Patel J.L., Smith L.M., Anderson J., Abromowitch M., Campana D., Jacobsen J., Perkins S.L. (2012). The immunophenotype of T‐lymphoblastic lymphoma in children and adolescents: a C hildren's O ncology G roup report. Br. J. Haematol..

[10] Pomari E., Lovisa F., Carraro E., Primerano S., D'Amore E.S., Bonvini P., Mussolin L. (2017). Clinical impact of miR-223 expression in pediatric T-Cell lymphoblastic lymphoma. Oncotarget.

[11] Burkhardt B., Moericke A., Klapper W., Greene F., Salzburg J., Damm-Welk C., Reiter A. (2008). Pediatric precursor T lymphoblastic leukemia and lymphoblastic lymphoma: differences in the common regions with loss of heterozygosity at chromosome 6q and their prognostic impact. Leuk. Lymphoma.

[12] López-Nieva P., Vaquero C., Fernández-Navarro P., González-Sánchez L., Villa-Morales M., Santos J., Fernández-Piqueras J. (2012). EPHA7, a new target gene for 6q deletion in T-cell lymphoblastic lymphomas. Carcinogenesis.

[13] Sinclair P.B., Sorour A., Martineau M., Harrison C.J., Mitchell W.A., O'Neill E., Foroni L. (2004). A fluorescence in situ hybridization map of 6q deletions in acute lymphocytic leukemia: identification and analysis of a candidate tumor suppressor gene. Cancer Res..

[14] Remke M., Pfister S., Kox C., Toedt G., Becker N., Benner A., Kulozik A.E. (2009). High-resolution genomic profiling of childhood T-ALL reveals frequent copy-number alterations affecting the TGF-β and PI3K-AKT pathways and deletions at 6q15-16.1 as a genomic marker for unfavorable early treatment response. J. Am. Soc. Hematol..

[15] Tabrizifard S., Olaru A., Plotkin J., Fallahi-Sichani M., Livak F., Petrie H.T. (2004). Analysis of transcription factor expression during discrete stages of postnatal thymocyte differentiation. J. Immunol..

[16] Massa S., Balciunaite G., Ceredig R., Rolink A.G. (2006). Critical role for c‐kit (CD117) in T cell lineage commitment and early thymocyte development in vitro. Eur. J. Immunol..

[17] Plum J., De Smedt M., Leclercq G., Verhasselt B., Vandekerckhove B. (1996).

[18] Delgado-Martin C., Meyer L.K., Huang B.J., Shimano K.A., Zinter M.S., Nguyen J.V., Hermiston M.L. (2017). JAK/STAT pathway inhibition overcomes IL7-induced glucocorticoid resistance in a subset of human T-cell acute lymphoblastic leukemias. Leukemia.

[19] Trinquand A., Tanguy-Schmidt A., Ben Abdelali R., Lambert J., Beldjord K., Lengliné E., Asnafi V. (2013). Toward a NOTCH1/FBXW7/RAS/PTEN-based oncogenetic risk classification of adult T-cell acute lymphoblastic leukemia: a Group for Research in Adult Acute Lymphoblastic Leukemia study. J. Clin. Oncol..

[20] Barata J.T., Silva A., Brandao J.G., Nadler L.M., Cardoso A.A., Boussiotis V.A. (2004). Activation of PI3K is indispensable for interleukin 7–mediated viability, proliferation, glucose use, and growth of T cell acute lymphoblastic leukemia cells. J. Exp. Med..

[21] Karawajew L., Ruppert V., Wuchter C., Kösser A., Schrappe M., Dörken B., Ludwig W.D. (2000). Inhibition of in vitro spontaneous apoptosis by IL-7 correlates with bcl-2 up-regulation, cortical/mature immunophenotype, and better early cytoreduction of childhood T-cell acute lymphoblastic leukemia. J. Am. Soc. Hematol..

[22] Oliveira M.L., Akkapeddi P., Ribeiro D., Melão A., Barata J.T. (2019). IL-7R-mediated signaling in T-cell acute lymphoblastic leukemia: an update. Adv. biol. regul..

[23] Burkhardt B., Mueller S., Khanam T., Perkins S.L. (2016). Current status and future directions of T-lymphoblastic lymphoma in children and adolescents. Br. J. Haematol..

[24] Zhang P., Zhang M. (2020 Nov 7). Epigenetic alterations and advancement of treatment in peripheral T-cell lymphoma. Clin. Epigenet..

[25] Ferenczi K., Makkar H.S. (2016). Cutaneous lymphoma: kids are not just little people. Clin. Dermatol..

[26] Trautinger F., Eder J., Assaf C. (2017). European Organisation for Research and Treatment of Cancer consensus recommendations for the treatment of mycosis fungoides/Sezary syndrome – update 2017. Eur. J. Cancer.

[27] Hwang S.T., Janik J.E., Jaffe E.S. (2008). Mycosis fungoides and Sezary syndrome. Lancet.

[28] Bagherani N, Smoller BR. An Overview of Cutaneous T Cell Lymphomas. F1000Res 2016;5:1882 PMID: 27540476 PMCID: PMC4965697 DOI: 10.12688/f1000research.8829.1. 10.12688/f1000research.8829.1 PMC4965697 27540476

[29] Kohnken R., Fabbro S., Hastings J. (2016). Sezary Syndrome: clinical and biological aspects. Curr. Hematol. Malig. Rep..

[30] Nicolay J.P., Felcht M., Schledzewski K. (2016). Sezary syndrome: old enigmas, new targets. J Dtsch Dermatol Ges.

[31] Laharanne E., Oumouhou N., Bonnet F. (2010). Genome-wide analysis of cutaneous T-cell lymphomas identifies three clinically relevant classes. J. Invest. Dermatol..

[32] Laharanne E., Chevret E., Idrissi Y. (2010). CDKN2A-CDKN2B deletion defines an aggressive subset of cutaneous T-cell lymphoma. Mod. Pathol..

[33] Salgado R., Servitje O., Gallardo F. (2010). Oligonucleotide array-CGH identifies genomic subgroups and prognostic markers for tumor stage mycosis fungoides. J. Invest. Dermatol..

[34] Choi J., Goh G., Walradt T. (2015). Genomic landscape of cutaneous T cell lymphoma. Nat. Genet..

[35] Harmon C.B., Witzig T.E., Katzmann J.A. (1996). Detection of circulating T cells with CD41CD7- immunophenotype in patients with benign and malignant lymphoproliferative dermatoses. J. Am. Acad. Dermatol..

[36] Jokinen C.H., Fromm J.R., Argenyi Z.B. (2011). Flow cytometric evaluation of skin biopsies for mycosis fungoides. Am. J. Dermatopathol..

[37] Ishitsuka K., Tamura K. (2014). Human T-cell leukemia virus type I and adult T-cell leukaemia-lymphoma. Lancet Oncol..

[38] Matsuoka M., Jeang K.T. (2007). Human T-cell leukaemia virus type 1 (HTLV-1) infectivity and cellular transformation. Nat. Rev. Cancer.

[39] Tsukasaki K., Hermine O., Bazarbachi A., Ratner L., Ramos J.C., Harrington W., O'Mahony D., Janik J.E., Bittencourt A.L., Taylor G.P., Yamaguchi K., Utsunomiya A., Tobinai K., Watanabe T. (2009). Definition, prognostic factors, treatment, and response criteria of adult T-cell leukemia-lymphoma: a proposal from an international consensus meeting. J. Clin. Oncol. : off. J. Am. Soc. Clinic. Oncol..

[40] Karube K., Aoki R., Sugita Y. (2008). The relationship of FOXP3 expression and clinicopathological characteristics in adult T-cell leukemia/lymphoma. Mod. Pathol..

[41] Sawada Y., Hino R., Hama K. (2011). Type of skin eruption is an independent prognostic indicator for adult T-cell leukemia/lymphoma. Blood.

[42] Matutes E. (2007). Adult T-cell leukaemia/lymphoma. J. Clin. Pathol..

[43] (1987). Fifth International Workshop on Chromosomes in Leukemia–lymphoma Correlation of chromosome abnormalities with histologic and immunologic characteristics in non‐Hodgkin's lymphoma and adult T‐cell leukemia lymphoma. Blood.

[44] Kataoka K., Nagata Y., Kitanaka A. (2015). Integrated molecular analysis of adult T cell leukemia/lymphoma. Nat. Genet..

[45] Campo E., Swerdlow S.H., Harris N.L., Pileri S., Stein H., Jaffe E.S. (2011). The 2008 WHO classification of lymphoid neoplasms and beyond: evolving concepts and practical applications. Blood.

[46] Harris N.L., Jaffe E.S., Stein H., Banks P.M., Chan J.K., Cleary M.L., Delsol G., De Wolf- Peeters C., Falini B., Gatter K.C. (1994). A revised European-American classification of lymphoid neoplasms: a proposal from the International Lymphoma Study Group [see comments]. Blood.

[47] Attygalle Ayoma, Al-Jehani Rajai, Tim C. (2002). Diss, phillipa munson, hongxiang liu, ming-qing du, peter G. Isaacson, ahmet dogan; neoplastic T cells in angioimmunoblastic T-cell lymphoma express CD10. Blood.

[48] Dogan A., Attygalle A.D., Kyriakou C. (2003). Angioimmunoblastic T-cell lymphoma. Br. J. Haematol..

[49] Laurence de Leval David S. Rickman, Thielen Caroline, de Reynies Aurélien, Huang Yen-Lin, Delsol Georges, Lamant Laurence, Leroy Karen, Brière Josette, Molina Thierry, Berger Françoise, Gisselbrecht Christian, Xerri Luc, Gaulard Philippe (2007). The gene expression profile of nodal peripheral T-cell lymphoma demonstrates a molecular link between angioimmunoblastic T-cell lymphoma (AITL) and follicular helper T (T_FH_) cells. Blood.

[50] Swerdlow S.H., Campo E., Pileri S.A., Harris N.L., Stein H., Siebert R., Advani R., Ghielmini M., Salles G.A., Zelenetz A.D., Jaffe E.S. (2016). The 2016 revision of the World Health Organization classification of lymphoid neoplasms. Blood.

[51] Odejide O., Weigert O., Lane A.A., Toscano D., Lunning M.A., Kopp N., Kim S., van Bodegom D., Bolla S., Schatz J.H., Teruya-Feldstein J., Hochberg E., Louissaint A., Dorfman D., Stevenson K., Rodig S.J., Piccaluga P.P., Jacobsen E., Pileri S.A., Harris N.L., Weinstock D.M. (2014). A targeted mutational landscape of angioimmunoblastic T-cell lymphoma. Blood.

[52] Li Z., Xia Y., Feng L.N., Chen J.R., Li H.M., Cui J., Cai Q.Q., Sim K.S., Nairismägi M.L., Laurensia Y., Meah W.Y., Liu W.S., Guo Y.M., Chen L.Z., Feng Q.S., Pang C.P., Chen L.J., Chew S.H., Ebstein R.P., Foo J.N., Liu J., Ha J., Khoo L.P., Chin S.T., Zeng Y.X., Aung T., Chowbay B., Diong C.P., Zhang F., Liu Y.H., Tang T., Tao M., Quek R., Mohamad F., Tan S.Y., Teh B.T., Ng S.B., Chng W.J., Ong C.K., Okada Y., Raychaudhuri S., Lim S.T., Tan W., Peng R.J., Khor C.C., Bei J.X. (2016 Sep). Genetic risk of extranodal natural killer T-cell lymphoma: a genome-wide association study. Lancet Oncol..

[53] Wang X., Gong Z., Li S.X., Yan W., Song Y. (2017 Sep 6). Extranodal nasal-type natural killer/T-cell lymphoma with penile involvement: a case report and review of the literature. BMC Urol..

[54] Dobashi A., Tsuyama N., Asaka R., Togashi Y., Ueda K., Sakata S., Baba S., Sakamoto K., Hatake K., Takeuchi K. (2016 May). Frequent BCOR aberrations in extranodal NK/T-Cell lymphoma, nasal type. Genes Chromosomes Cancer.

[55] Tse E., Kwong Y.L. (2013 Jun 20). How I treat NK/T-cell lymphomas. Blood.

[56] Kohrt H., Advani R. (2009 Nov). Extranodal natural killer/T-cell lymphoma: current concepts in biology and treatment. Leuk. Lymphoma.

[57] Wong K.F., Zhang Y.M., Chan J.K. (1999 Jul). Cytogenetic abnormalities in natural killer cell lymphoma/leukaemia--is there a consistent pattern?. Leuk. Lymphoma.

[58] Tse E., Kwong Y.L. (2017 Apr 14). The diagnosis and management of NK/T-cell lymphomas. J. Hematol. Oncol..

[59] Sakajiri S., Kawamata N., Egashira M., Mori K., Oshimi K. (2001 Oct). Molecular analysis of tumor suppressor genes, Rb, p53, p16INK4A, p15INK4B and p14ARF in natural killer cell neoplasms. Jpn. J. Cancer Res..

[60] Siu L.L., Chan J.K., Wong K.F., Choy C., Kwong Y.L. (2003 Jul). Aberrant promoter CpG methylation as a molecular marker for disease monitoring in natural killer cell lymphomas. Br. J. Haematol..

[61] Iqbal J., Weisenburger D.D., Chowdhury A., Tsai M.Y., Srivastava G., Greiner T.C., Kucuk C., Deffenbacher K., Vose J., Smith L., Au W.Y., Nakamura S., Seto M., Delabie J., Berger F., Loong F., Ko Y.H., Sng I., Liu X., Loughran T.P., Armitage J., Chan W.C. (2011 Feb). International Peripheral T-cell Lymphoma Project. Natural killer cell lymphoma shares strikingly similar molecular features with a group of non-hepatosplenic γδ T-cell lymphoma and is highly sensitive to a novel aurora kinase A inhibitor in vitro. Leukemia.

[62] Ng S.B., Selvarajan V., Huang G., Zhou J., Feldman A.L., Law M., Kwong Y.L., Shimizu N., Kagami Y., Aozasa K., Salto-Tellez M., Chng W.J. (2011 Mar). Activated oncogenic pathways and therapeutic targets in extranodal nasal-type NK/T cell lymphoma revealed by gene expression profiling. J. Pathol..

[63] (2000 Sep). The world health organization classification of malignant lymphomas in Japan: incidence of recently recognized entities. Lymphoma Study Group of Japanese Pathologists. Pathol. Int..

[64] Isaacson P.G., Du M.Q. (2005 Jan). Gastrointestinal lymphoma: where morphology meets molecular biology. J. Pathol..

[65] Okumura K., Ikebe M., Shimokama T., Takeshita M., Kinjo N., Sugimachi K., Higashi H. (2012 May 21). An unusual enteropathy-associated T-cell lymphoma with MYC translocation arising in a Japanese patient: a case report. World J. Gastroenterol..

[66] Ondrejka S., Jagadeesh D. (2016 Dec). Enteropathy-associated T-cell lymphoma. Curr. Hematol. Malig. Rep..

[67] Ferreri A.J., Zinzani P.L., Govi S., Pileri S.A. (2011 Jul). Enteropathy-associated T-cell lymphoma. Crit. Rev. Oncol. Hematol..

[68] Deleeuw R.J., Zettl A., Klinker E., Haralambieva E., Trottier M., Chari R., Ge Y., Gascoyne R.D., Chott A., Müller-Hermelink H.K., Lam W.L. (2007 May). Whole-genome analysis and HLA genotyping of enteropathy-type T-cell lymphoma reveals 2 distinct lymphoma subtypes. Gastroenterology.

[69] Baumgärtner A.K., Zettl A., Chott A., Ott G., Müller-Hermelink H.K., Starostik P. (2003 Oct). High frequency of genetic aberrations in enteropathy-type T-cell lymphoma. Lab. Invest..

[70] Al Somali Z., Hamadani M., Kharfan-Dabaja M., Sureda A., El Fakih R., Aljurf M. (2021 Apr). Enteropathy-associated T cell lymphoma. Curr. Hematol. Malig. Rep..

[71] Tomita S., Kikuti Y.Y., Carreras J., Kojima M., Ando K., Takasaki H., Sakai R., Takata K., Yoshino T., Bea S., Campo E., Nakamura N. (2015 Oct). Genomic and immunohistochemical profiles of enteropathy-associated T-cell lymphoma in Japan. Mod. Pathol..

[72] Lamant L., Meggetto F., al Saati T. (1996). High incidence of the t(2;5)(p23;q35) translocation in anaplastic large cell lymphoma and its lack of detection in Hodgkin's disease. Comparison of cytogenetic analysis, reverse transcriptase-polymerase chain reaction, and P-80 immunostaining. Blood.

[73] Mason D.Y., Bastard C., Rimokh R. (1990;74(February). CD30-positive large cell lymphomas (‘Ki-1 lymphoma’) are associated with a chromosomal translocation involving 5q35. Br. J. Haematol..

[74] Morris S.W., Kirstein M.N., Valentine M.B. (1994). Fusion of a kinase gene, ALK, to a nucleolar protein gene, NPM, in non-Hodgkin’s lymphoma. Science.

[75] Brousset P., Rochaix P., Chittal S. (1993). High incidence of EpsteinBarr virus detection in Hodgkin's disease and absence of detection in anaplastic large-cell lymphoma in children. Histopathology.

[76] Salaverria I., Beà S., Lopez-Guillermo A. (2008). Genomic profiling reveals different genetic aberrations in systemic ALK-positive and ALK-negative anaplastic large cell lymphomas. Br. J. Haematol..

[77] Piva R., Agnelli L., Pellegrino E. (2010). Gene expression profiling uncovers molecular classifiers for the recognition of anaplastic largecell lymphoma within peripheral T-cell neoplasms. J. Clin. Oncol..

[78] Bonzheim I., Geissinger E., Roth S., Zettl A., Marx A., Rosenwald A., Rüdiger T. (2004). Anaplastic large cell lymphomas lack the expression of T-cell receptor molecules or molecules of proximal T-cell receptor signaling. Blood.

[79] Feldman E.J. (2011). Too much ara-C? Not enough daunorubicin?. J. Am. Soc. Hematol..

[80] Luchtel R.A., Zimmermann M.T., Hu G. (2019). Recurrent MSC (E116K) mutations in ALK-negative anaplastic large cell lymphoma. Blood.

[81] Xing X., Feldman A.L. (2015). Anaplastic large cell lymphomas: ALK positive, ALK negative, and primary cutaneous. Adv. Anat. Pathol..

[82] Broccoli A., Zinzani P.L. (2017). Peripheral T-cell lymphoma, not otherwise specified. Blood.

[83] Foss Francine M., Zinzani Pier Luigi, Vose Julie M., Gascoyne Randy D., Rosen Steven T. (2011). Kensei tobinai; peripheral T-cell lymphoma. Blood.

[84] Al-Zahrani M., Savage K.J. (2017). Peripheral T-cell lymphoma, not otherwise specified: a review of current disease understanding and therapeutic approaches. Hematol. Oncol. Clin. N. Am..

[85] Schmitz N., de Leval L. (2017). How I manage peripheral T-cell lymphoma, not otherwise specified and angioimmunoblastic T-cell lymphoma: current practice and a glimpse into the future. Br. J. Haematol..

[86] Maura F. (2016). Biology of peripheral T cell lymphomas—not otherwise specified: is something finally happening?. Pathogenesis.

[87] Sakata-Yanagimoto M., Chiba S. (2015). Molecular pathogenesis of peripheral T cell lymphoma. Curr. hematol. malig. rep..

[88] Swerdlow S.H., Campo E., Pileri S.A., Harris N.L., Stein H., Siebert R., Advani R., Ghielmini M., Salles G.A., Zelenetz A.D., Jaffe E.S. (2016). The 2016 revision of the World Health Organization classification of lymphoid neoplasms. Blood.

[89] de Leval Laurence, Gaulard Philippe (2014). Cellular origin of T-cell lymphomas. Blood.

[90] Zain J.M., Hanona P. (2021). Aggressive T-cell lymphomas: 2021 Updates on diagnosis, risk stratification and management [published correction appears in Am J Hematol. 2021 Jul 22. Am. J. Hematol..

[91] Weiner D.M., Durgin J.S., Wysocka M., Rook A.H. (2021). The immunopathogenesis and immunotherapy of cutaneous T cell lymphoma: current and future approaches. J. Am. Acad. Dermatol..

[92] Sweetenham J.W. (2009). Treatment of lymphoblastic lymphoma in adults. Oncology (Williston Park).

